# Investigating the Relationship Between Visual Evoked Potentials, Neurological and Neuropsychological Status in Primary Progressive Multiple Sclerosis

**DOI:** 10.3390/jcm15135031

**Published:** 2026-06-28

**Authors:** Jasna Duranović, Sanda Pavelin, Vanna Žnidar, Ivana Miloš, Krešimir Dolić, Joško Šoda, Maja Rogić Vidaković

**Affiliations:** 1 Laboratory for Human and Experimental Neurophysiology (LAHEN), Department of Neuroscience, School of Medicine, University of Split, 21000 Split, Croatia; jasna.duranovic@mefst.hr; 2 Department of Neurology, University Hospital of Split, 21000 Split, Croatia; sanda.pavelin@mefst.hr; 3 Department of Neurology, School of Medicine, University of Split, 21000 Split, Croatia; 4 Department of Medical Biology, School of Medicine, University of Split, 21000 Split, Croatia; 5 Department of Physiology, School of Medicine, University of Split, 21000 Split, Croatia; ivana.milos@mefst.hr; 6 Department of Interventional and Diagnostic Radiology, University Hospital of Split, 21000 Split, Croatia; kdolic@kbsplit.hr; 7 Department of Radiology, School of Medicine, University of Split, 21000 Split, Croatia; 8 Faculty of Health Sciences, University of Split, 21000 Split, Croatia; 9 Department of Nanoelectronics and Photovoltaics, Faculty of Electrical Engineering, Mechanical Engineering and Naval Architecture, University of Split, 21000 Split, Croatia; jsoda@fesb.hr

**Keywords:** primary progressive multiple sclerosis, neurophysiology, visual evoked potentials, EDSS, neuropsychology

## Abstract

**Background/Objectives**: Visual evoked potential (VEP) P100 maximum latency is known to predict fatigue and cognition in multiple sclerosis (MS). However, the relationship between VEP and neurological (impairment)/neuropsychological status is not well understood in MS. Therefore, the aim of the present prospective cross-sectional study was to investigate associations among VEP, neurological impairment, and neuropsychological status in MS. **Methods**: The study included 25 subjects with primary progressive MS (PPMS), with a median EDSS score of 4.5, and 30 control subjects. Neurological status of PPMS subjects was evaluated with the Expanded Disability Status Scale (EDSS), while neuropsychological assessment included the Symbol Digit Modalities Test (SDMT), Nine-Hole Peg Test (9HPT), Fatigue Severity Scale (FSS), Depression, Anxiety and Stress Scale (DASS-21), and Multiple Sclerosis Impact Scale (MSIS-29). Statistical analysis included group comparisons, Spearman correlations, multivariable linear regression analyses, bidirectional stepwise selection, and a parsimonious multivariable model. **Results**: PPMS subjects had significantly prolonged P100 maximum latencies (*p* < 0.01) compared to control subjects, as well as significantly poorer performance on the 9HPT (*p* < 0.01) and higher fatigue scores (FSS; *p* < 0.01). In multivariable analyses within the PPMS group, disease duration was independently associated with the EDSS (*p* = 0.009), whereas neuropsychological measures were intercorrelated (ρ > 0.45) and associated with the EDSS at intermediate stages of model selection. P100 maximum latency showed a positive but non-significant association after adjustment for sex and disease duration in the parsimonious model, which explained approximately 49% of the variance in the EDSS. **Conclusions**: The findings provide insight into the complex interplay among neurophysiological (VEP), neuropsychological and neurological status in MS.

## 1. Introduction

Multiple sclerosis (MS) is a chronic, inflammatory, and demyelinating autoimmune disease affecting the central nervous system (CNS). It is the most prevalent non-traumatic disabling illness among adults, impacting more than 2.8 million people globally [1,2]. As for the course of the disease, MS is classified as either relapsing-remitting (RRMS) or progressive (PMS). RRMS is the predominant clinical subtype, affecting approximately 85–90% of MS individuals. Patients diagnosed with RRMS experience acute neurological symptoms during relapses, which are subsequently followed by remissions. Over time, individuals with RRMS may transition to secondary progressive multiple sclerosis (SPMS), marked by a continuous decline in functional ability, which may occur with or without the presence of relapses. Primary progressive MS (PPMS) accounts for 10–15% of cases and is characterised by a gradual deterioration in neurological function, typically occurring without distinct relapses [3].

From a pathological perspective, MS is identified by diverse multifocal lesions within the CNS, such as the optic nerve, brain regions, or the spinal cord [4,5]. The lesions include chronic lesions, gliotic or demyelinated scars, acute inflammatory damage to the myelin, oligodendrocytes, axons, or lesions with reduced inflammation and preserved structures [3]. The key drivers for the development of focal lesions and clinical relapses are T and B lymphocytes [6,7,8], with macrophages and monocytes contributing to both proinflammatory and anti-inflammatory responses [9,10]. The clinical characteristics of MS vary based on the affected CNS regions. The interrelation between visual, sensory, and motor pathways leads to diverse clinical symptoms like muscle weakness, sensory deficits, cognitive impairment, and fatigue [11].

The diagnosis of MS is established through a combination of laboratory analyses, in accordance with the 2024 McDonald criteria and the 2021 MAGNIMS-CMSC-NAIMS guidelines [5,12], including the identification of distinct bands of immunoglobulins representative of individual lymphocyte populations in the cerebrospinal fluid and magnetic resonance imaging (MRI) performed at a field strength of ≥1.5 T, which reveals T2 lesions in the brain and spinal cord [5,12]. The MS neurological status is evaluated by the Expanded Disability Status Scale (EDSS) [13], assessing seven functional systems (plus ambulation). In addition to impairment evaluation, other measures are often used, such as the Nine-Hole Peg Test (9HPT), applied for finger dexterity exploration [14,15,16,17], and the Symbol Digit Modalities Test (SDMT), assessing cognitive information processing speed [18,19,20,21,22,23]. Similarly, positive associations among fatigue, depression, anxiety and sleep quality have been found in MS [24], as well as their positive associations with the EDSS [24,25,26]. Despite standard clinical tools for diagnosing MS, the relationship between pathophysiological mechanisms and clinical manifestations remains insufficiently understood, highlighting potential relevance of subclinical measures in MS (blood markers, evoked potentials or neuropsychological assessment scores) [27,28,29,30,31,32,33,34,35,36,37,38,39,40,41,42,43,44,45,46,47,48,49,50,51,52,53]. Altered visual evoked potential (VEP) latency and connectivity of visual pathways to different cortical regions have been associated with global cognitive dysfunction in PPMS [54,55], supporting the notion that VEPs may be useful as a biomarker of cortical/connectivity abnormalities beyond the visual system in MS [54]. The visual pathway is connected to downstream regions that support higher-level cognition; therefore, VEPs may measure cortical activity and neural efficiency during perception and cognition [56]. Further, fatigue, depression and anxiety are associated with altered recruitment of neural resources, network dysfunction, and disruptions in pathways involving basal ganglia, frontal cortex, thalamus, amygdala and hippocampus in MS [57,58,59,60,61]. VEP [62,63] can index brain activity and assess disturbances linked to cognitive impairment and physical disability in MS [64,65]. Prolonged P100 latency correlates with longer disease duration and damage in the retina and in the optic radiation [66]. Moreover, VEP amplitude and latency deteriorate in MS subjects with higher EDSS scores [67]. Further, prolonged P100 latency correlates with poorer performance in multiple cognitive domains [54,68] and with a two-year decline in information processing speed [56]. Also, prolonged VEP latency significantly predicts global and cognitive fatigue over a two-year period in MS subjects [69].

Even though VEP might provide complementary information about neurological impairment and cognitive status [64,65,70], the relationship among neurophysiological, neurological, and neuropsychological measures in PPMS remains insufficiently understood. Previous studies predominantly included heterogeneous MS populations and incomplete reporting of EDSS or disease duration information [54,56,69,70,71], small sample of PPMS subjects [54,56,69,70,71,72], limited comparison of data with control subjects [54,56,69,70,71], and applied non-standardised neuropsychological measures [29,37,54,56,69,70,71]. These considerations are particularly relevant in PPMS, where continuous disease progression without relapse-related fluctuations may provide a more stable framework for examining associations between neurophysiological markers, cognitive performance, and neurological impairment [73,74]. Due to its lower prevalence compared with RRMS, studies focused specifically on PPMS are less common and evidence regarding neurophysiological and neuropsychological correlates in this population remains limited [75].

To better understand the relationship between neurological impairment and subclinical measures, the present prospective study aims to investigate the relationships between neurological (EDSS), neurophysiological (VEP), and neuropsychological status (information processing speed, finger dexterity, fatigue, depression, anxiety, stress, physical and psychological impact of MS disease) in PPMS.

## 2. Materials and Methods

### 2.1. Participants

The prospective study included a total of 25 PPMS subjects (15 treated with ocrelizumab and 10 untreated, mean age of 56.88 ± 8.40 years, 72% female) and 30 control subjects matched by age, sex, height, and education level. The criterion for inclusion in the study was the absence of clinical or neuroradiological disease activity for at least three months before assessment. The exclusion criteria were the presence of comorbidities affecting ambulation; history of diseases of the central or peripheral nervous system (other than MS); history of psychiatric diseases; or history of drug or alcohol abuse.

### 2.2. Study Protocol

PPMS subjects underwent neurological examination, followed by VEP and neuropsychological testing on the same day.

**Figure 1:**
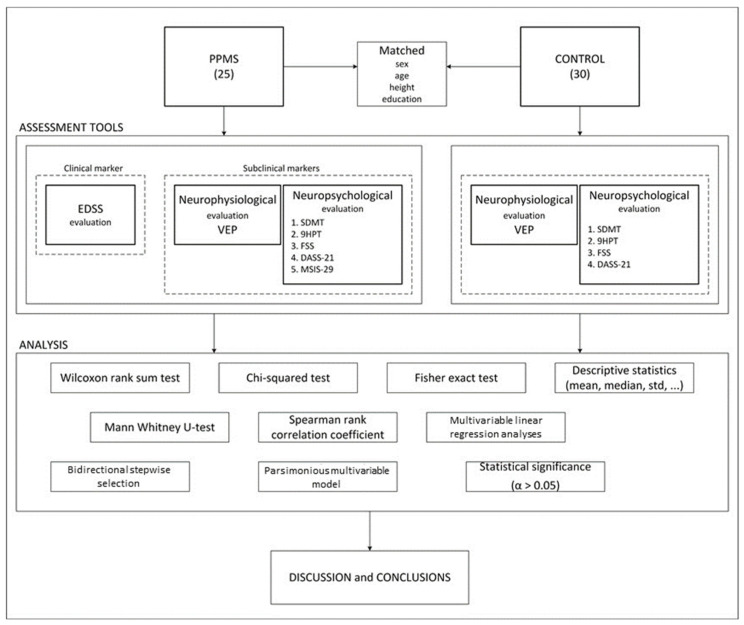
Framework of the study. Abbreviations: EDSS—Expanded Disability Status Scale; SDMT—Symbol Digit Modalities Test; 9HPT—Nine-Hole Peg Test; FSS—Fatigue Severity Scale; DASS-21—Depression, Anxiety and Stress Scale; MSIS-29—Multiple Sclerosis Impact Scale.

Figure 1 shows the framework of the study comparing PPMS subjects (*N* = 25) with control subjects (*N* = 30), matched by sex, age, height and education. For assessment, the EDSS and subclinical measures (VEP and neuropsychological status) were evaluated.

### 2.3. Visual Evoked Potentials

Neurophysiological VEP examination was conducted using Neuropack^®^ X1 MEB-2300 (Nihon Kohden Corporation, Tokyo, Japan). Preparation of the skin included cleaning with ethanol and an abrasive gel (SkinPure YZ-0019, Nihon Kohden Corporation, Tokyo, Japan) to ensure skin-electrode contact impedance of 5 kΩ or less. The electrodes were placed according to the international 10–20 system. The recording electrode was placed on the visual cortex (Oz), with a reference electrode on Fz and a ground electrode on Cz using conductive gel (Elefix V ZV-181E, Nihon Kohden Corporation, Tokyo, Japan). Recording and reference electrodes were obtained from the NE-132B Evoked EEG Electrode Set by Nihon Kohden Corporation (Tokyo, Japan). The stimulation (a full-field chessboard pattern) was presented to one eye with the other closed (Opticlude™ orthoptic eye patch with two cotton pads underneath) in a quiet and dimly lit room. The subject was asked to focus on the white square in the middle of the screen while remaining relaxed and awake. Regarding stimulation parameters, contrast polarity was alternated at a frequency of 1 Hz, and the contrast of the chessboard pattern was modulated to maintain the visual stimulus with a pattern size of 32. The stimulation duration was continuous. The high-pass filter was set to 100 Hz, the low-pass filter to 1 Hz, and the number of averaged signals was 100. Display mode was set to reverse mode, and pattern display time was 100 ms. For each eye, latency and amplitude for N75, P100, and N145 waves were obtained.

### 2.4. Neurological and Neuropsychological Evaluation

Neurological examination for PPMS subjects involved determining disease duration and assessing impairment using EDSS score evaluation, which was conducted by a qualified neurologist (author of the study) [13,76]. The EDSS evaluates eight functional systems: pyramidal, cerebellar, brain stem, sensory, bowel and bladder, visual, cerebral and ambulation. The scale is scored in an ordinal format that ranges from 0 (indicating a normal neurological exam) to 10.0 (representing death due to MS), with increments of 0.5 points [13].

The neuropsychological evaluation was performed by a qualified psychologist (author of the study), who administered neuropsychological tests including the Symbol Digit Modalities Test (SDMT) and the Nine-Hole Peg Test (9HPT) [15,29,37,77]. Also, subjects completed the following patient-derived measures: the Fatigue Severity Scale (FSS), the Depression, Anxiety and Stress Scale (DASS-21), and the Multiple Sclerosis Impact Scale (MSIS-29) [37,51]. The control subjects did not fill out the MSIS-29, as it is a disease-specific measure not applicable to healthy controls.

#### 2.4.1. Symbol Digit Modalities Test (SDMT)

The Symbol Digit Modalities Test (SDMT) [77] evaluates processing speed and sustained attention by mainly focusing on intricate visual scanning and tracking tasks. To ensure consistent administration across study participants, the written version of the SDMT was used in all assessments, where the subject is presented with a page featuring a key that matches the single digits from 1 to 9 with corresponding symbols. Below the key, there are rows filled solely with symbols, and the subject’s task is to write down the correct number in the designated spaces. After the subject completes the initial 10 items with assistance, their performance is timed to see how many responses they can generate within 90 s. Overall, the test requires a maximum of 5 min to complete [77,78].

#### 2.4.2. Nine-Hole Peg Test (9HPT)

The Nine-Hole Peg Test (9HPT) [15,79] measures finger dexterity. Testing was conducted using the Jamar 9-Hole Peg Test Kit (ASP Global, LLC, Austell, GA, USA). The 9HPT consists of a square board featuring nine pegs. To administer the test, subjects are instructed to sequentially take pegs from a container and insert them into the holes on the board as quickly as possible. After completing this task, subjects must remove the pegs from the holes one at a time and return them to the container. The test is conducted using both the dominant and non-dominant hands. Subjects are evaluated based on the time taken to complete the task, recorded in seconds [79,80].

#### 2.4.3. Fatigue Severity Scale (FSS)

The Fatigue Severity Scale (FSS) [81] is a self-assessment questionnaire consisting of 9 items designed to evaluate fatigue experienced over the past week by individuals with multiple sclerosis (MS). Each item is scored on a scale from 1 to 7, where 1 reflects complete disagreement and 7 indicates strong agreement. The overall score is determined by summing all responses and dividing by nine to obtain an average. A cut-off score of 4 or higher is considered indicative of clinically significant fatigue [82]. The Croatian version of the FSS for MS subjects was used [83].

#### 2.4.4. Depression, Anxiety and Stress Scale (DASS-21)

The Depression, Anxiety and Stress Scale (DASS-21) [84] is a self-report scale that represents a shortened version of the original DASS-42 questionnaire and consists of a total of 21 statements distributed in three subscales with an equal number of items: depression, anxiety, and stress on a four-point Likert scale. For each statement, subjects are asked to circle the number in the column that best describes how they felt in the last week, from 0—did not apply to me at all to 3—applied to me very much or most of the time. The result on each subscale is expressed as the sum of the estimates on the corresponding items [84]. The Croatian version of DASS-21 for individuals with MS was used [51].

#### 2.4.5. Multiple Sclerosis Impact Scale (MSIS-29)

The Multiple Sclerosis Impact Scale (MSIS-29) [39] is a self-assessment tool designed to evaluate the effects of multiple sclerosis on patients’ physical and psychological function. It comprises two subscales: the physical subscale, which has 20 items focusing on physical effects, and the psychological subscale, which contains 9 items assessing psychological effects. Each question asks for the subject’s opinion about the impact of MS over the past two weeks by circling a number on a five-point Likert scale, where 1 means “not at all” and 5 means “extremely”. The overall score for each subscale is calculated by summing the items and converting them into a score out of 100 [39]. The Croatian adaptation of the MSIS-29 scale was used [51].

### 2.5. Statistical Analysis

For VEP, latency (measured in milliseconds) and amplitude (measured in microvolts) were obtained from N75, P100, and N145 waves. The maximum P100 latency (the longer of the two P100 latencies obtained for the left and right eyes) was analysed as a measure of effective index variance in neural activity in MS subjects [54].

In the analysis of sociodemographic and disease-related variables, the Wilcoxon rank-sum test was employed for continuous variables, while the Chi-squared test or Fisher’s exact test was used for categorical variables, depending on the distribution and sample size. Descriptive statistics were presented as percentages, means, standard deviations, medians, or interquartile ranges for all relevant subject characteristics and applied psychometric scales. Group differences were assessed using the Mann–Whitney U test. To explore associations between variables of interest, bivariate correlations were calculated using Spearman’s rank correlation coefficient. To examine associations between the EDSS and neuropsychological/neurophysiological variables within the PPMS group, multivariable linear regression analyses were performed (Supplementary Materials, Tables S2–S7). Given the relatively small sample size and the number of candidate predictors, multicollinearity was assessed using variance inflation factors (VIFs), with values > 5 considered indicative of substantial collinearity. As an exploratory approach to reduce model complexity, bidirectional stepwise selection based on the Akaike Information Criterion (AIC) was applied, starting from a base model including age, sex, and BMI. Because stepwise procedures may lead to overfitting in small samples, these results were interpreted as exploratory and were further evaluated for residual multicollinearity. To obtain a stable and clinically interpretable model, a reduced multivariable model excluding highly collinear predictors was fitted. Finally, a parsimonious multivariable model including sex and disease duration, with P100 maximum latency retained as the primary neurophysiological variable of interest, was constructed to assess its association with the EDSS.

An alpha level of 0.05 was used to determine statistical significance [85,86]. All statistical analyses were conducted using R 4.4.2. [87].

## 3. Results

Table 1 presents the basic sociodemographic characteristics and disease-related information of all subjects. In the group of subjects diagnosed with PPMS, the mean age was 56.88 ± 8.40 years, and 72% were female. The median EDSS score was 4.5 with an interquartile range of 2.00, and an average disease duration of 8.71 ± 8.34 years. The most prevalent comorbid conditions within the PPMS group involved disorders of the circulatory (20.00%) and endocrine system (20.00%). In the control group, the subjects had an average age of 54.8 ± 8.00 years. The most prevalent comorbidity for the control group was related to the circulatory system (36.67%).

**Figure 2:**
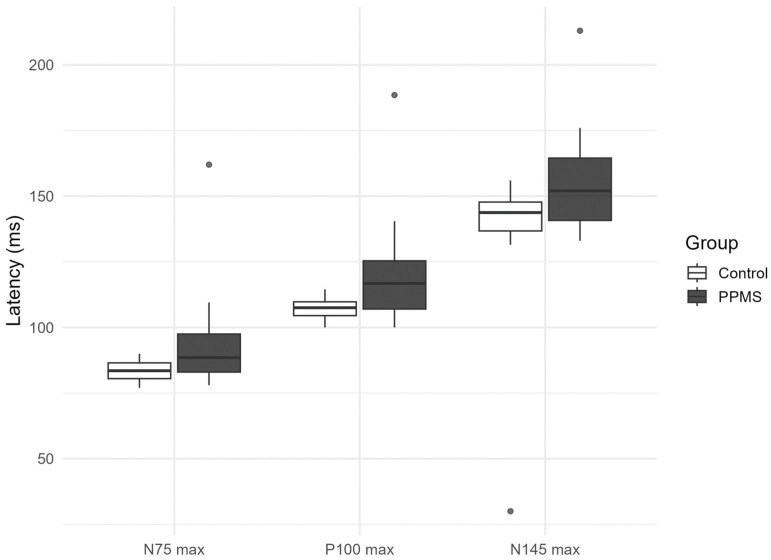
Box plots highlighting between-group differences in N75, P100, and N145 maximum latencies. Legend: N75 max—VEP N75 maximum latency, P100 max—VEP P100 maximum latency, N145 max—VEP N145 maximum latency.

Group differences were analysed across all variables of interest. PPMS subjects were found to have significantly prolonged N75 (U = 136.00, *p* = 0.02), P100 (U = 163.00, *p* < 0.01) and N145 (U = 117.00, *p* = 0.02) maximum latencies, compared to control subjects. PPMS subjects demonstrated significant impairment on the 9HPT (U = 119.00, *p* < 0.01) and FSS (U = 180.00, *p* < 0.01). Table 2 presents between-group comparisons in VEP and neuropsychological measures, whereas Figure 2 presents box plots highlighting N75, P100, and N145 maximum latencies of the two-sample non-parametric comparisons.

Table S1 in the Supplementary Materials presents Spearman correlation coefficients among the EDSS, VEP latencies, and neuropsychological status for PPMS and control subjects, respectively.

Strong positive associations were found between fatigue and DASS-21 depression (ρ = 0.69), DASS-21 anxiety (ρ = 0.54), DASS-21 stress (ρ = 0.45), MSIS-29 physical (ρ = 0.73) and MSIS-29 psychological impacts of MS (ρ = 0.63) in PPMS. Additionally, a strong negative correlation exists between SDMT and 9HPT (ρ = −0.66) scores. Other significant correlations observed in PPMS subjects include a positive relationship between the EDSS and disease duration (ρ = 0.53). Furthermore, correlations between physical impact of MS and depression (ρ = 0.63), anxiety (ρ = 0.48) and stress (ρ = 0.51) were found, as well as between psychological impact of MS and depression (ρ = 0.76), anxiety (ρ = 0.61) and stress (ρ = 0.78). On the other hand, in the control group, the most prominent correlations are the positive correlation between the SDMT and education level (ρ = 0.51) and the negative correlation between the SDMT and 9HPT (ρ = −0.54). No associations between VEP variables and neuropsychological measures were found in either group (*p* > 0.05).

To examine associations between the EDSS and neuropsychological/neurophysiological variables within the PPMS group, bidirectional stepwise AIC model selection and multivariable linear regression analysis were performed (Supplementary Materials, Tables S2–S7). A parsimonious multivariable prediction model including sex and disease duration was fitted, with P100 latency retained as the primary neurophysiological variable of interest. In this model, P100 latency showed a positive, although non-significant, association with the EDSS (Table 3 and Table 4).

## 4. Discussion

The present study revealed intergroup differences between PPMS and control subjects in VEP N75, P100 and N145 maximum latencies, FSS, and 9HPT. Consistent with the study aim to explore associations between neurological status and subclinical measures, multivariable analyses were conducted, incorporating the EDSS, VEP P100 maximum latency, neuropsychological, and sociodemographic variables. Initial exploratory models suggested that several patient-reported outcome measures were strongly interrelated, resulting in substantial multicollinearity. After model refinement, disease duration emerged as the primary factor associated with the EDSS. P100 maximum latency showed a positive association with impairment; however, this relationship did not reach statistical significance in the final parsimonious prediction model.

The study findings are consistent with previous studies highlighting prolonged P100 maximum latencies, as well as higher levels of fatigue and poorer finger dexterity [66,88,89,90] in PPMS. Furthermore, the predictive value of P100 maximum latency has been previously confirmed in MS studies exploring the relationship between P100 latency and neuropsychological aspects of MS, primarily fatigue and cognitive functioning domains [54,56,69,70,71]. Prolonged P100 maximum latency is associated with impaired performance in information processing speed, executive function, attention, and motor skills, global cognitive performance, as well as global and cognitive fatigue [54,56,69]. The present study indicates that P100 maximum latency has a positive, non-significant association with the EDSS in PPMS, while no association was found between P100 maximum latency and neuropsychological variables. The finding on positive non-significant association of the EDSS with P100 maximum latency aligns with the limitations of the EDSS, which, at higher score ranges (especially from EDSS 4.0 onwards), predominantly reflects walking ability, while the contribution of visual function is relatively small [13,24,91].

In the present study, P100 maximum latency was used as a predictor in regression models in MS, building upon the VEP study findings of Covey et al. [54,56,69,70,71]. While prior studies investigated different aspects of fatigue and cognitive factors as dependent variables, EDSS scores were not incorporated into the regression models. Therefore, the present study contributes to the existing literature through the examination of the association between P100 maximum latency and neurological status (EDSS) within a multivariable framework accounting for sociodemographic and neuropsychological factors. In addition, intercorrelations were observed among neuropsychological measures, more precisely a moderate negative correlation between the SDMT and 9HPT scores, a moderate positive correlation between the FSS and DASS-21 subscales, and moderate-to-high positive correlations between the DASS-21 and MSIS-29 subscales. The findings are consistent with recent reports on stronger intercorrelations among non-motor symptoms (fatigue, depression, anxiety, stress, physical and psychological impact of MS) in MS, and weaker associations of these non-motor symptoms with neurological status (EDSS) [24]. Our results extend previous findings [24] by showing a moderately negative association between manual dexterity and information processing speed, suggesting a specific cognitive-motor interaction that complements the correlations among non-motor symptoms in MS [92].

While the present findings offer a unique perspective into the interrelationships of the VEP, EDSS, and neuropsychological parameters, several limitations should be considered when interpreting the results. Constraints of the study are related to the smaller PPMS sample size recruited from a single centre, which might elucidate the nonsignificant results regarding the between-group differences in information processing speed and mental health indicators (depression, anxiety, stress) [44,93,94]. Moreover, caution is necessary when drawing conclusions on sex differences in the PPMS sample, as this study included 25 PPMS subjects, 72% of whom were female. The multivariable findings should be interpreted in the context of the relatively smaller sample size. Although model reduction strategies and assessment of multicollinearity were implemented to improve model stability and interpretability, the possibility of overfitting cannot be entirely excluded. Replication in larger cohorts is warranted to assess the robustness of the observed associations. In addition, although a written version of the SDMT was used in this study, future studies with PPMS populations may consider using the oral SDMT to reduce the potential effects of upper extremity motor impairment on test performance [78,95].

Nonetheless, this study included control subjects matched by sex and age to PPMS and assessed neuropsychological status using validated measures (SDMT, 9HPT, FSS, DASS-21, MSIS-29) [29,37,96], addressing a limitation of a recent study [70]. Hence, future studies should include a larger sample of PPMS subjects, particularly male PPMS subjects, and consider including other neurophysiological tools, other than VEP, such as somatosensory evoked potential (SEP) and motor evoked potential (MEP) [38,97], along with assessments of the EDSS and neuropsychological status.

## 5. Conclusions

The findings of the present study suggest a complex relationship among the EDSS, VEP, and neuropsychological status in PPMS, providing a unique perspective on their interrelationships. VEP P100 maximum latency showed a positive, although non-significant, relationship with the EDSS in PPMS, whereas neuropsychological measures were intercorrelated and associated with the EDSS at intermediate stages of model selection. Overall, the observed associations among the EDSS, VEP, and neuropsychological status in PPMS indicate that neurophysiological and neuropsychological measures reflect distinct aspects of neurological impairment, and therefore may contribute to a more comprehensive understanding of PPMS [38,71,97].

## Figures and Tables

**Table 1:** Sociodemographic and disease-related data of PPMS and control subjects.

	PPMS (*N* = 25)	Controls (*N* = 30)	*p*
Age (years), M ± SD	56.88 ± 8.40	54.83 ± 8.00	0.63
Sex Male, *n* (*%*) Female, *n* (*%*)	7 (28.00%) 18 (72.00%)	12 (40.00%) 18 (60.00%)	0.52
BMI, M ± SD	25.88 ± 3.66	26.30 ± 3.94	0.69
Education Elementary school, *n* (*%*) High school, *n* (*%*) Undergraduate studies, *n* (*%*) Graduate studies, *n* (*%*) Doctorate studies, *n* (*%*)	4 (16.00%) 16 (64.00%) 3 (12.00%) 2 (8.00%) 0 (0.00%)	1 (3.33%) 17 (56.67%) 4 (13.33%) 6 (20.00%) 2 (6.67%)	0.28
Work status Employed, *n* (*%*) Unemployed, *n* (*%*) Currently on sick leave, *n* (*%*) Retired, *n* (*%*)	8 (33.33%) 4 (16.67%) 2 (8.33%) 10 (41.67%)	21 (70.00%) 5 (16.67%) 0 (0.00%) 4 (13.33%)	0.02
Marital status Single, *n* (*%*) Married, *n* (*%*) In a common-law relationship, *n* (*%*) Divorced, *n* (*%*) Widowed, *n* (*%*)	3 (12.00%) 19 (76.00%) 0 (0.00%) 2 (8.00%) 1 (4.00%)	2 (6.67%) 25 (83.33%) 1 (3.33%) 1 (3.33%) 1 (3.33%)	0.86
Diopter, *n* has prescription (*%*)	21 (84.00%)	21 (70.00%)	0.34
Handedness, *n* left-handed (*%*)	0 (0.00%)	3 (10.00%)	0.24
Comorbidities			0.19
Diseases of the circulatory system, *n* (*%*)	5 (20.00%)	11 (36.67%)	
Endocrine, nutritional, and metabolic diseases, *n* (*%*)	5 (20.00%)	4 (13.33%)	
Diseases of the respiratory system, *n* (*%*)	1 (4.00%)	2 (6.67%)	
Diseases of the musculoskeletal system and connective tissue, *n* (*%*)	2 (8.00%)	9 (30.00%)	
Diseases of the blood and blood-forming organs and certain disorders involving the immune mechanism, *n* (*%*)	0 (0.00%)	1 (3.33%)	
Diseases of the digestive system, *n* (*%*)	1 (4.00%)	0 (0.00%)	
Diseases of the genitourinary system, *n* (*%*)	1 (4.00%)	0 (0.00%)	
Diseases of the skin and subcutaneous tissue, *n* (*%*)	0 (0.00%)	2 (6.67%)	
EDSS, median [Q1–Q3]	4.50 [3.0–5.0]		
Disease duration (years), M ± SD	8.71 ± 8.34		

Abbreviations: M—mean; SD—standard deviation; BMI—Body Mass Index; Q1—first quartile; Q3—third quartile; EDSS—Expanded Disability Status Scale. EDSS and disease duration represent disease-related measures.

**Table 2:** Descriptive data on VEP and neuropsychological variables and rank-based group comparisons.

	PPMS (*N* = 25)	Controls (*N* = 30)	U	*p*
Left-eye N75 latency (ms), M ± SD	91.36 ± 19.86	82.40 ± 4.83	179.00	0.10
Left-eye N75 amplitude (µV), M ± SD	2.44 ± 2.14	2.10 ± 1.93	225.00	0.73
Left-eye P100 latency (ms), M ± SD	117.21 ± 18.80	107.29 ± 3.90	183.00	0.03
Left-eye P100 amplitude (µV), M ± SD	−2.16 ± 2.33	−3.58 ± 4.19	117.00	0.07
Left-eye N145 latency (ms), M ± SD	152.71 ± 21.76	140.58 ± 6.95	173.00	0.07
Left-eye N145 amplitude (µV), M ± SD	4.63 ± 3.60	5.02 ± 4.11	238.00	0.76
Right-eye N75 latency (ms), M ± SD	88.69 ± 12.89	82.17 ± 3.89	167.50	0.04
Right-eye N75 amplitude (µV), M ± SD	2.31 ± 1.66	2.39 ± 1.99	242.50	0.84
Right-eye P100 latency (ms), M ± SD	112.99 ± 13.01	106.87 ± 4.05	214.00	0.09
Right-eye P100 amplitude (µV), M ± SD	−2.26 ± 2.58	−4.69 ± 3.53	113.50	0.02
Right-eye N145 latency (ms), M ± SD	144.99 ± 14.31	140.59 ± 8.08	204.00	0.38
Right-eye N145 amplitude (µV), M ± SD	4.15 ± 3.05	4.64 ± 3.59	193.00	0.37
Maximum N75 latency (ms), M ± SD	93.35 ± 18.26	83.72 ± 3.87	136.00	0.02
Maximum P100 latency (ms), M ± SD	119.18 ± 18.17	107.07 ± 4.28	163.00	<0.01
Maximum N145 latency (ms), M ± SD	155.74 ± 19.22	138.34 ± 25.21	117.00	0.02
SDMT score, M ± SD	37.30 ± 16.30	42.13 ± 10.73	277.50	0.23
9HPT score (s), M ± SD	30.81 ± 12.36	22.75 ± 3.04	119.00	<0.01
FSS score, M ± SD	4.71 ± 1.63	3.29 ± 1.54	180.00	<0.01
DASS-21 depression score, M ± SD	4.63 ± 4.62	2.70 ± 3.02	254.00	0.06
DASS-21 anxiety score, M ± SD	4.13 ± 4.31	3.23 ± 3.19	324.00	0.53
DASS-21 stress score, M ± SD	7.00 ± 4.41	6.07 ± 4.78	308.00	0.37
MSIS-29 physical impact score, M ± SD	35.94 ± 19.20			
MSIS-29 psychological impact score, M ± SD	29.05 ± 22.21			

Abbreviations: M—mean; SD—standard deviation; SDMT—Symbol Digit Modalities Test; 9HPT—Nine-Hole Peg Test; FSS—Fatigue Severity Scale; DASS-21—Depression, Anxiety and Stress Scale; MSIS-29—Multiple Sclerosis Impact Scale.

**Table 3:** Parsimonious multivariable linear regression model of the EDSS in the PPMS group.

Model:	EDSS = sex + disease duration + P100 maximum latency			
Min	1Q	Median	3Q	Max
−2.59	−0.50	0.15	0.80	1.33
Coefficients	Estimate	Std. Error	t	*p*
Intercept	1.65	1.76	0.94	0.360
Sex	−0.91	0.57	−1.59	0.13
Disease duration	0.10	0.03	2.94	0.009
P100 maximum latency	0.02	0.02	1.31	0.208
RSE = 1.13, df_residual_ = 16				
R^2^ = 0.49, R_adj_^2^ = 0.39				
F(3,16) = 5.03, *p* = 0.012				

Abbreviations: EDSS—Expanded Disability Status Scale.

**Table 4:** Variance inflation factors (VIFs) for the parsimonious multivariable model.

	VIF
Sex	1.09
Disease duration	1.08
P100 maximum latency	1.02

## Data Availability

The data that support the findings of this study are available upon request from the corresponding author. The data are not publicly available due to privacy or ethical restrictions.

## Supplementary Materials

The following supporting information can be downloaded at: https://www.mdpi.com/article/10.3390/jcm15135031/s1, Table S1: Spearman correlation coefficients between EDSS, VEP latencies and neuropsychological status for PPMS and control subjects; Table S2: Variance inflation factors (VIF) for candidate predictors in the initial multivariable model; Table S3: Bidirectional stepwise AIC model selection (PPMS group) – model selection summary; Table S4: Bidirectional stepwise AIC model selection (PPMS group) – Final stepwise model coefficients; Table S5: Variance inflation factors (VIF) for predictors retained in the final stepwise AIC model; Table S6: Reduced multivariable model after multicollinearity adjustment (PPMS group); Table S7: Variance inflation factors (VIF) for the reduced multivariable model.

## Abbreviations

The following abbreviations are used in this manuscript:

AIC	Akaike Information Criterion
CNS	Central nervous system
DASS-21	Depression, Anxiety and Stress Scale
EDSS	Expanded Disability Status Scale
FSS	Fatigue Severity Scale
MEP	Motor evoked potential
MS	Multiple sclerosis
MSIS-29	Multiple Sclerosis Impact Scale
9HPT	Nine-Hole Peg Test
PPMS	Primary progressive multiple sclerosis
RRMS	Relapsing-remitting multiple sclerosis
SDMT	Symbol Digit Modalities Test
SEP	Somatosensory evoked potential
VIF	Variance inflation factor
VEP	Visual evoked potential
