# Supplementary material for: Investigating the Relationship Between Visual Evoked Potentials, Neurological and Neuropsychological Status in Primary Progressive Multiple Sclerosis
Source: J Clin Med. 2026 Jun 28;15(13):5031. doi: 10.3390/jcm15135031 (PMC13362041; doi:10.3390/jcm15135031)
Supplement: Supplementary file 1 [file jcm-15-05031-s001.zip › jcm-4347767-supplementary.pdf]

Supplementary Materials

Table S1. Spearman correlation coefficients between EDSS, VEP latencies and neuropsychological status for PPMS and control subjects.

	Group	*EDSS*	*Disease duration*	*SDMT*	*9HPT*	*FSS*	*DASS-21 (depression)*	*DASS-21 (anxiety)*	*DASS-21 (stress)*	*MSIS-29 PHYS*	*MSIS-29 PSY*	*N75 maximum latency*	*P100 maximum latency*	*N145 maximum latency*
EDSS	1	-	-	-	-	-	-	-	-	-	-	-	-	-
Disease duration	1	**0.53**	-	-	-	-	-	-	-	-	-	-	-	-
SDMT	1	-0.35	-0.14	-	-	-	-	-	-	-	-	-	-	-
9HPT	1	0.18	0.17	**-0.66**	-	-	-	-	-	-	-	-	-	-
FSS	1	**0.41**	**0.57**	0.08	0.16	-	-	-	-	-	-	-	-	-
DASS- 21 (depression)	1	0.04	0.06	-0.05	0.31	**0.69**	-	-	-	-	-	-	-	-
DASS-21 (anxiety)	1	0.03	0.09	0.38	-0.05	**0.54**	**0.59**	-	-	-	-	-	-	-
DASS-21 (stress)	1	-0.20	0.01	0.26	0.08	**0.45**	**0.71**	**0.72**	-	-	-	-	-	-
MSIS-29 PHYS	1	0.38	**0.52**	-0.13	0.29	**0.73**	**0.63**	**0.48**	**0.51**	-	-	-	-	-
MSIS-29 PSY	1	-0.04	0.21	0.24	0.04	**0.63**	**0.76**	**0.61**	**0.78**	**0.73**	-	-	-	-
N75 maximum latency	1	0.30	0.17	-0.16	0.25	0.21	0.04	0.05	-0.13	0.07	0.07	-	-	-
P100 maximum latency	1	0.11	0.03	0.25	0.13	0.08	-0.14	0.08	-0.04	-0.02	0.01	**0.76**	-	-

N145 maximum latency	1	-0.03	0.10	0.21	0.35	0.09	0.00	0.02	0.10	0.11	0.28	**0.48**	**0.82**	-
EDSS	2	-	-	-	-	-	-	-	-	-	-	-	-	-
Disease duration	2	-	-	-	-	-	-	-	-	-	-	-	-	-
SDMT	2	-	-	-	-	-	-	-	-	-	-	-	-	-
9HPT	2	-	-	**-0.54**	-	-	-	-	-	-	-	-	-	-
FSS	2	-	-	-0.11	0.01	-	-	-	-	-	-	-	-	-
DASS- 21 (depression)	2	-	-	-0.07	0.04	0.33	-	-	-	-	-	-	-	-
DASS-21 (anxiety)	2	-	-	-0.05	-0.16	0.30	**0.60**	-	-	-	-	-	-	-
DASS-21 (stress)	2	-	-	0.10	-0.15	0.11	**0.69**	**0.79**	-	-	-	-	-	-
MSIS-29 PHYS	2	-	-	-	-	-	-	-	-	-	-	-	-	-
MSIS-29 PSY	2	-	-	-	-	-	-	-	-	-	-	-	-	-
N75 maximum latency	2	-	-	-0.23	0.22	0.09	-0.07	**-0.44**	-0.29	-	-	-	-	-
P100 maximum latency	2	-	-	-0.17	0.02	0.17	0.15	-0.16	-0.15	-	-	0.35	-	-
N145 maximum latency	2	-	-	-0.25	0.37	0.04	0.02	-0.14	-0.01	-	-	0.22	**0.57**	-

Note: Group 1 – PPMS subjects; Group 2 – control subjects

Abbreviations: EDSS - Expanded Disability Status Scale; SDMT – Symbol Digit Modalities Test; 9HPT – Nine-hole Peg Test; FSS – Fatigue Severity Scale; DASS-21 – Depression, Anxiety and Stress Scale; MSIS-29 PHYS - Physical subscale of MSIS-29; MSIS-29 PSY - Psychological subscale of MSIS-29.

Table S2. Variance inflation factors (VIF) for candidate predictors in the initial multivariable model.

	VIF
Age	3.41
Sex	2.18
BMI	1.85
Disease duration	4.73
FSS	7.18
DASS-21 (depression)	10.73
DASS-21 (anxiety)	4.49
DASS-21 (stress)	7.80
MSIS-29 PHYS	7.36
MSIS-29 PSY	10.13
SDMT	2.79
9HPT	2.67
P100 maximum latency	1.66

Abbreviations: EDSS - Expanded Disability Status Scale; BMI - Body Mass Index; SDMT – Symbol Digit Modalities Test; 9HPT – Nine-hole Peg Test; FSS – Fatigue Severity Scale; DASS-21 – Depression, Anxiety and Stress Scale; MSIS PHYS - Physical subscale of MSIS-29; MSIS PSY - Psychological subscale of MSIS-29.

Table S3. Bidirectional stepwise AIC model selection (PPMS group) – model selection summary.

Step	Model	AIC
Initial model	EDSS ~ Age + Sex + BMI	16.61
Final stepwise model	EDSS ~ Age + Sex + BMI + P100 maximum latency + MSIS-29 PSY + MSIS-29 PHYS + DASS-21 (anxiety) + disease duration + DASS-21 (stress) + 9HPT	-3.64

Abbreviations: EDSS - Expanded Disability Status Scale; BMI - Body Mass Index; 9HPT – Nine-hole Peg Test;; DASS-21 – Depression, Anxiety and Stress Scale; MSIS PHYS - Physical subscale of MSIS-29; MSIS PSY - Psychological subscale of MSIS-29.

Table S4. Bidirectional stepwise AIC model selection (PPMS group) – Final stepwise model coefficients.

Predictor	*β*	*Std. Error*	*t*	*p*
Age	-0.04	0.03	-1.17	.273
Sex	-1.73	0.46	-3.76	.004
BMI	-0.07	0.07	-1.05	.319
P100 maximum latency	0.04	0.01	3.14	.012
MSIS-29 PSY	-0.08	0.02	-3.39	.008
MSIS-29 PHYS	0.07	0.02	3.44	.007
DASS-21 (anxiety)	0.13	0.07	1.82	.102
Disease duration	0.08	0.03	2.64	.027
DASS-21 (stress)	0.15	0.10	1.50	.169
9HPT	-0.03	0.03	-1.15	.281

RSE = 0.79

$R^2 = 0.86$, $R_{adj}^2 = 0.71$

$F(10,9) = 5.54$, $p = .008$

Abbreviations: EDSS - Expanded Disability Status Scale; BMI - Body Mass Index; 9HPT – Nine-hole Peg Test; DASS-21 – Depression, Anxiety and Stress Scale; MSIS PHYS - Physical subscale of MSIS-29; MSIS PSY - Psychological subscale of MSIS-29.

Table S5. Variance inflation factors (VIF) for predictors retained in the final stepwise AIC model.

	VIF
Age	1.71
Sex	1.44
BMI	1.81
P100 maximum latency	1.42
MSIS PSY	8.73
MSIS PHYS	5.10
DASS-21 (anxiety)	3.22
Disease duration	2.05
DASS-21 (stress)	6.28
9HPT	1.92

Abbreviations: EDSS - Expanded Disability Status Scale; BMI - Body Mass Index; 9HPT – Nine-hole Peg Test; DASS-21 – Depression, Anxiety and Stress Scale; MSIS PHYS - Physical subscale of MSIS-29; MSIS PSY - Psychological subscale of MSIS-29.

Table S6. Reduced multivariable model after multicollinearity adjustment (PPMS group).

Model:	EDSS ~ age + sex + BMI + Disease duration + P100 maximum latency + DASS-21 (anxiety) + 9HPT			
Min	*1Q*	*Median*	*3Q*	*Max*
-2.25	-0.45	0.07	0.61	1.30
Coefficients	*Estimate*	*Std. Error*	*t*	*p*
Intercept	2.52	3.61	0.70	.498
Age	-0.02	0.05	-0.44	.667
Sex	-1.42	0.63	-2.27	.043*
BMI	-0.03	0.08	-0.38	.711
Disease duration	0.09	0.04	2.31	.039*
P100 maximum latency	0.03	0.02	1.66	.122
DASS-21 (anxiety)	0.14	0.07	2.01	0.07
9HPT	-0.00	0.04	-0.11	.912
RSE=1.11, $df_{residual}$=12<br>R^2=0.63, R_{adj}^2=0.41<br>F(7,12)=2.87, p=.052				

Abbreviations: EDSS - Expanded Disability Status Scale; BMI - Body Mass Index; 9HPT – Nine-hole Peg Test; DASS-21 – Depression, Anxiety and Stress Scale.

Table S7. Variance inflation factors (VIF) for the reduced multivariable model.

	VIF
Age	1.63
Sex	1.34
BMI	1.40
Disease duration	1.66
P100 maximum latency	1.32
DASS-21 anxiety	1.56
9HPT	1.60

Abbreviations: EDSS - Expanded Disability Status Scale; BMI - Body Mass Index; 9HPT – Nine-hole Peg Test; DASS-21 – Depression, Anxiety and Stress Scale.
